# A Therapeutic Potential for Marine Skeletal Proteins in Bone Regeneration

**DOI:** 10.3390/md11041203

**Published:** 2013-04-10

**Authors:** David W. Green, Matthew P. Padula, Jerran Santos, Joshua Chou, Bruce Milthorpe, Besim Ben-Nissan

**Affiliations:** 1 Department of Chemistry and Forensic Science, University of Technology Sydney, Ultimo, NSW 2007, Australia; E-Mails: joshua.chou@uts.edu.au (J.C.); bruce.milthorpe@uts.edu.au (B.M.); besim.ben-nissan@uts.edu.au (B.B.-N.); 2 Proteomics Core Facility, Faculty of Science, University of Technology Sydney, Ultimo, NSW 2007, Australia; E-Mails: matthew.padula@uts.edu.au (M.P.P.); jerran.naidoo-2@uts.edu.au (J.S.)

**Keywords:** proteomics, bone tissue engineering, mesenchymal stem cells, marine invertebrate skeletons, bone matrix proteins

## Abstract

A vital ingredient for engineering bone tissue, in the culture dish, is the use of recombinant matrix and growth proteins to help accelerate the growth of cultivated tissues into clinically acceptable quantities. The skeletal organic matrices of calcifying marine invertebrates are an untouched potential source of such growth inducing proteins. They have the advantage of being ready-made and retain the native state of the original protein. Striking evidence shows that skeleton building bone morphogenic protein-2/4 (BMP) and transforming growth factor beta (TGF-β) exist within various marine invertebrates such as, corals. Best practice mariculture and the latest innovations in long-term marine invertebrate cell cultivation can be implemented to ensure that these proteins are produced sustainably and supplied continuously. This also guarantees that coral reef habitats are not damaged during the collection of specimens. Potential proteins for bone repair, either extracted from the skeleton or derived from cultivated tissues, can be identified, evaluated and retrieved using chromatography, cell assays and proteomic methods. Due to the current evidence for bone matrix protein analogues in marine invertebrates, together with the methods established for their production and retrieval there is a genuine prospect that they can be used to regenerate living bone for potential clinical use.

## 1. Introduction

### Key Issues in Regenerative Orthopaedics

Regenerative orthopaedics is the science of building and growing large amounts of natural human musculoskeletal tissue in the culture dish, with an emphasis on using stem cell precursors. Entire living bone tissue replacements are not available in clinical practice because the quantities of tissue, needed in oral, maxillofacial and orthopaedic surgeries, cannot be faithfully reproduced [1]. There is another problem too, as the composition, anatomical structure and final function of culture-derived tissue does not accurately simulate the human archetype. To do this properly requires a support framework with features of an extracellular matrix, proteins to control development and potentiated cell types that re-assemble into tissues. As of now, scaffold-based tissue engineering is providing many useful structural environments where tissues can be reconstituted in their natural form and with normal functions [2,3,4,5,6]. However, there are two outstanding issues that need to be addressed if tissues are to be regenerated fully in the culture dish. The first is to recreate a blood system within the developing tissue and provide adequate nutrition. The second is to simulate the delivery schedule of developmental proteins to cells for proliferation and differentiation into whole tissues. So far, clinical trials implementing these factors, in the regeneration of tissues, have not led to the anticipated results, because it has not been possible to target the proteins at the correct physiological dosages and in a well-timed sequence. To ensure that enough natural quality bone is produced in the laboratory, bone regeneration must, ideally start with the recreation of an integrated cellular and molecular ecosystem made up of blood vessels, neurons, cells and regenerative biochemicals. This is vital for the proper simulation of tissue development derived from cell progenitors [7]. Stem cells are the ideal choice to begin tissue regeneration. Because tissue engineers have not been able to successfully assemble such a difficult composite of elements, there has been a shift in approach towards the fabrication of materials and structures containing proteins, which are dispersed in controlled ways, to orchestrate endogenous repair, remodeling and regeneration [8]. The addition of growth promoting proteins is the key to driving and orchestrating tissue regeneration in the culture dish. Tissue promoting proteins used in experimental and clinical regenerative therapies are expensive to produce. The production of proteins using recombinant technology is imperfect. Recombinant proteins do not have the molecular modifications that are introduced during normal, routine genetic processing by native human cells. This has made it difficult to make genuine native proteins with their entire set of evolved functions. Thus, there are good scientific reasons for developing relatively straightforward, low cost alternatives. Marine invertebrates are one potential, an unexamined source of select proteins with potential utility in strategies for regenerative medicine, in the laboratory, and possibly for the patient. We shall explain why this is a feasible and convincing option to follow. To give this message strength and clarity, it is important to highlight the fact that proteins have been retained during the progressive increments in evolutionary history. There are also some persuasive examples where proteins can interchange between evolutionary distant organisms.

## 2. The Earliest Proteins and Genes Have Been Safeguarded throughout Animal Evolution

Less intricate organisms at the base of the evolutionary tree such as, marine invertebrates may seem an unlikely resource for the bone tissue engineer, but they display a significant richness and diversity of intact frameworks, metabolic products, enzymes, signaling proteins, glycosaminoglycans, sterol lipids, extracellular matrix components and structural biomaterials that can usefully function in human physiology [9,10,11]. Many of these factors listed have almost identical functions in marine invertebrates and humans. This is supported by data that shows the physiological functions of some ancient proteins, operating in lower vertebrates, are identical to the human version of the protein, despite the existence of structural and compositional differences [12]. 

**Figure 1:**
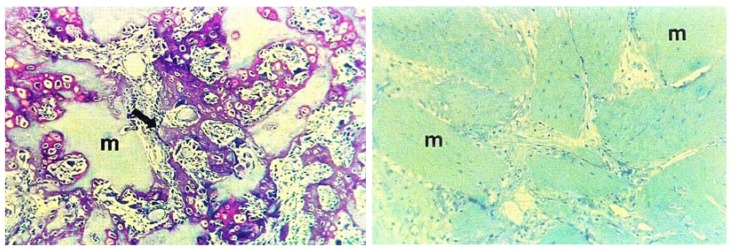
Bone formation in mammals is made possible with a bone inductive protein analogue derived from an invertebrate. In this example, a protein from Drosophila development and an analogue of skeleton building bone morphogenic protein-2/4 (BMP), was injected into the muscle of a mouse, generating new bone. The histological section on the left shows osteoinduction in living mouse subcutaneous tissue following treatment with Drosophila Decapentaplegic (dpp). New bone tissue has been reproduced shown in purple. On the right, for comparison is a histological section of untreated mouse subcutaneous tissue. (Reproduced with permission from PNAS, Sampath *et al.* 1993 [13]).

Nature is a consummate innovator but key innovations are often preserved rather than completely re-designed. In one instance, insulin from Carp has been used to treat diabetes in humans. It is recognized as being the closest analogue of any other animal derived insulin including pig. The amino acid sequence of insulin, in some invertebrates, is similar to human insulin and regulates physiological levels of sugars in the human body [12]. Developmental proteins are some of the earliest proteins in evolution and are strongly preserved. Their roles have been selected time and time again. The embryonic pattern forming proteins, Decapentaplegic (dpp) and 60A are generated in *Drosophila*. Sections of the protein are identical to vertebrate skeleton building bone morphogenic protein-2/4 (BMP’s). The amino acid sequence of this section shares 70%–75% similarity with human BMP2/4 and 5/6 respectively. This is mirrored in the function of the protein as well. When concentrated preparations of dpp were injected into rat muscle they spontaneously invoked bone formation (Figure 1) [13].

Marine derived glycosaminoglycans (GAG) such as, Heparin sulphate are already being advocated as safer replacements for human obtained GAGs [10]. Although, marine invertebrate tissues are the simplest in composition and structural organization in the animal kingdom, they harness the biomolecular tool kit for the most basic and essential physiological processes and functions that exist in more highly evolved human tissues. To fully comprehend the molecular and structural similarities present between marine invertebrates and humans we need to compare and contrast all the sets of gene products and proteins.

Marine invertebrates evolved a larger than at first pre-supposed genome by natural selection. It was always considered that the size of a genome was related to the complexity of an organism. Their genome is involved in producing a large number of gene duplicates, which generate a quantity of refined protein alternatives. Sequencing of a marine sponge genome has shown notable congruence with other animal genomes [14]. This molecular reservoir of functional proteins in marine dwelling invertebrates supports the sizeable chemical diversity of secondary metabolites to out-compete peers, stifle predators and adapt dynamically to changes in environment. Sequence data from marine invertebrate genomes, such as corals, will ensure that clear comparisons can be made of the similarities to human genes [15]. Identified similarities will show which gene controlled processes and functions that the marine invertebrate and humans have in common. This will also require analysis of the transcriptome using the latest RNA sequencing methods [16]. Knowing the detail of the organism’s genetic constitution and what genes are switched on and off during biological processes tells us what to expect about the degree of richness of other molecules that arise from the genetic programming. Additional study and molecular manipulation is required to determine when these genes are turned on, how they are regulated and how much they express. We must be also sufficiently thorough in our gene analysis to describe splice variants and isoforms, because these actions modify the protein structures and functions. Proteins are the key machine parts that make biological processes work properly, as they have evolved to do so, and fit the purpose. So, to understand the functional outcomes of marine invertebrate gene activities it is necessary to accurately catalogue the proteins that are made by them. This is done using tools developed by scientists engaged in proteomics.

## 3. Preservation of Proteins during the Evolutionary History of Biomineralization

The level of molecular complementarity between invertebrates and humans is brought into focus by tracking the stepwise developments during the evolution of skeletal biomineralisation from its inception in the Cambrian, around 500 million years ago. Mineralisation in organisms evolved many times independently during this geological period. This strongly implies that the molecular elements at the heart of skeletal calcification-signal transmitters, inhibitors, promoters, capacitors, transcription factors in organisms during this period of rapid evolution were favored by natural selection pressures. This led to the adoption of the same pioneering molecular mechanisms in every calcifying organism since, throughout the evolution of biomineralization [17]. These basic machine components for the mineralization of biological tissues are found in, corals, sponges, molluscs and vertebrates, with slight modifications added stepwise with each evolutionary innovation. Together they undertake the fundamental procedures for mineralizing the organic skeletal template. 

There must be a strong possibility that key proteins, already linked to all calcifying animals are paired to those that exact fundamental tasks in the different stages of human bone mineralization-within bone matrices—such as, bone morphogenic proteins and osteonectin [18,19]. Some of the growth promoting molecules, principally peptides, used in the early stages of skeletal development have been discovered, already. A good example is the presence of an analogue to human osteonectin—alternatively named, SPARC found in species of marine sponge and Cnidarians. The osteonectin from these marine invertebrates possessed the same basic molecular organization and functions, in extracellular matrix adhesion, as their human equivalent [18,19]. This is not an isolated example. The rules of molecular evolution in organisms dictate with regularity, preservation of molecules rather than continued evolution of new ones. So, there will be other instances where proteins will have emerged by evolutionary innovations within developing lineages and clades.

**Figure 2:**
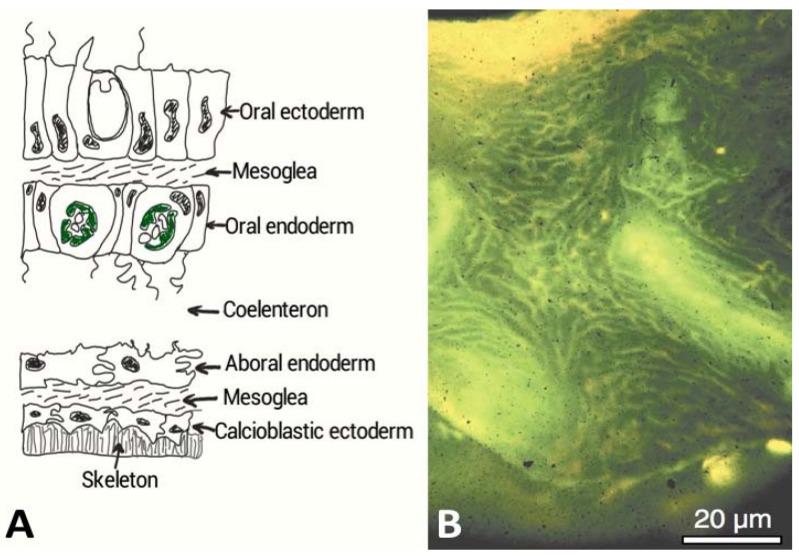
(**A**) A cross-sectional diagram through the living tissue portion of a coral and showing the interface with the exoskeleton. The calcioblastic epithelium secretes the organic matrix probably embedded within intracellular vesicles into micron or nanometric sized spaces. Biological control of mineralization is strongly implicated in corals in other ways. There are semi-permeable tight septate junctions between the cells that control ion transport and other molecules according to size and charge (After Allemand *et al.* [22]). The calcioblastic epithelium effectively facilitates the laying down of the inorganic skeleton; (**B**) Acridine orange staining of the organic matrix of *Acropora* sp. skeleton, which appears strong yellow at the growing region and green deeper inside the skeleton. The pale yellow regions are the centres of calcification. This microscope image was taken under polarized light and shows the global distribution of intra-skeletal organic matrices throughout the entire skeleton [25]. The coral tissue has been removed to view this organic matrix. (Reproduced with permission from the Institute of Paleobiology, Polish Academy of Sciences, Gautret *et al.* 2000 [25]).

There are other unique examples where identical keystone developmental proteins occur between primitive and advanced organisms. So for instance, the blood vessel forming VEGF and its complementary VEGF-Receptor are present in jellyfish [20]. Bone regeneration proteins BMP2/4 prevalent in vertebrate bone chemistry are also found in marine invertebrates such as, corals [21]. Within coral skeletons, BMP2/4 have been implicated in increasing organic matrix production by calcioblastic epithelial cells and its secretion into the compartmentalized calcifying medium (Figure 2A) [21]. BMP2/4 has been localized using immunohistochemistry in the calciodermis, which is the outward facing epithelium. Corals possess a rich organic matrix composition with probable regenerative, osteoactive, calcification and ECM proteins. They also harbor lipids, polysaccharides and glycosaminoglycans (GAGs). All these molecules are embedded within skeletal and extracellular organic matrices (e.g., mesoglea, desmocytes and historical matrix secretions within the bulk skeleton) that participate in skeletal morphogenesis [22]. Ensembles of proteins are secreted by corals within the extracellular matrix and into the skeleton at the mineralizing front by epidermal cells (Figure 2A). The distribution occurs extensively as rivulets throughout the coral skeleton (Figure 2B).

The secretion of growth factors into the skeleton occurs throughout adult life to offset frequent tissue loss and damage due to predation, disease and dynamic changes in their physical and chemical environment. Corals have a strong capacity to properly and fully regenerate their skeletons, following tissue degradation and damage and so have the skeletal proteins, for reconstruction, continually available [23]. Cell-to-cell signaling networks that are vital for the construction and development of calcified tissue in vertebrate bone, Wnt, TGF-β, notch and hedgehog are also used in the same way by calcareous marine sponges [24]. 

## 4.Marine Bioprospecting for Skeletal Matrix Proteins

Many marine organisms are enriched in what we now know to be a wealth of unique molecules that participate in development, metabolism and defense. There is also likely to be undiscovered molecules as well. Sessile marine organisms with highly developed chemical defences, have been systematically exploited as sources of potent secondary metabolite drug compounds [26]. However, the availability of such metabolites is severely limited and they are too intricate to convert into synthetic analogues. So far, yields of these clinically relevant chemical compounds are predominantly too low to be of any practical medical use. Other, non-metabolic structural and morphogenic proteins are likely to be more prevalent.

Making use of whole organisms as providers of useful biomedicines is subject to many restrictions. The collection of organisms from the wild is labor intensive, time consuming and costly to carry out. The collection is made more difficult because the distribution of candidate marine invertebrates can often be patchy. Also, the mass of tissues from a selected species is usually too low to justify the effort in harvesting [27]. There is also a considerable risk that once interesting therapeutic biomolecules are retrieved from the chosen marine invertebrate there may not be sufficient amounts for commercial scale development, processing and clinical use [27]. Often these therapeutic chemical compounds may also only be present in organisms growing in specific environmental conditions [28]. These facts have dissuaded pharmaceutical companies to abandon their natural product harvesting programs. At another level—marine conservation—it is important to select candidate marine organisms having a wide geographical distribution and low ecological significance, so that collecting them does not degrade levels of biodiversity. There are effective ways of tackling these problems such as, by rearing the marine organism in dedicated culture tanks. To qualify, the organism must have relatively high growth rates, fast wound healing rates and low disease susceptibility [29]. Alternatively, it may be practically feasible to select, enrich and artificially cultivate marine invertebrate cells that specifically secrete organic matrix, mineral bound, and extracellular matrix proteins of interest [30]. It is then possible to tailor the growth conditions, with a high degree of control, to promote increased secretion of desired molecules and install multiple millions of cells into tanks of growing media. The technological expertise is available and whole organisms can be farmed viably inside protected *ex situ* coastal nurseries [31,32] dissuading pharmaceutical companies to abandon their natural product harvesting programs. 

A significant number of marine invertebrates produce biochemicals within extracellular matrices and connective tissues, with like-for-like molecular structures, building block sequences and functions to human versions [18,19]. On this basis, it is therefore hypothesized that skeletal proteins in biomineral growth, maintenance and repair could offer the current tissue engineer a repertoire of proteins—with human physiological activity—that can help accelerate lab-based bone morphogenesis and increase bone volumes with equivalent potency to current recombinant proteins, or perhaps exceed them. The biggest obstacle to success will be to find ways of producing the desired protein in large amounts. Recombinant methods do not work well with some genes and do not have the capacity to modify the protein structure and composition in the same way as non-bacterial, eukaryocyte cells do. Using engineered expression vectors to overproduce the protein is feasible when they are small in size. Skeletal proteins with regenerative potency may also be used to complement treatments with such recombinant proteins. 

## 5. Marine Invertebrates Are Sources of Proteins for Regenerative Orthopaedics

We proceed to investigate how discovering and retrieving key developmental proteins in skeletal calcification, from marine invertebrates might proceed and how the extracted proteins might be used as candidates for replacements and adjuncts to existing growth promoting proteins, currently used in the production of clinically acceptable therapeutic human bone. Since marine organisms have evolved, by natural selection, a significant molecular resource for building, adapting and repairing calcified skeletal tissues, they could in-turn provide a clinically acceptable cluster of proteins that help accelerate the growth of human bone from precursor cells in tissue culture and within the patient’s own skeleton. 

There is published data to show that essential growth factor proteins function within selected marine invertebrate tissues. These are identical to growth factors present within human mineralized tissues. Three orders of marine invertebrates—corals, molluscs and marine sponges—have been most studied in this regard (Table 1). However, we still do not know the total number and complete set of proteins within any marine invertebrate. Still there has been partial characterization of the amino acid composition and molecular weights of proteins for selected coral and marine sponge extracellular organic matrices (Table 2 and Table 3). These do give hints, simplistically, to protein identities via there molecular weights alone. 

**Table 1:** Growth factor protein equivalents: A list of principal growth factors used in regenerative orthopaedics and their marine invertebrate equivalents.

Growth factor in bone matrix	Normal role in bone	Marine invertebrate species with growth factor analogue	Normal role in selected marine invertebrate
Bone morphogenic protein (BMP-2, BMP-4)	A keystone regulator of embryonic pattern formation. A key regulator of bone induction, maintenance and repair [33].	Corals: *Turbinaria reniformis*, *Acropora* sp.,* Pavona cactus*, *Galaxea fascicularis*, *Hydnophora pilosa*, *Stylophora pistillata*, *Lobophyllia* sp. [21,34].	Secreted by calcifying epithelium during adult skeletogenesis [35]. Larva and polyp axial patterning in embryogenesis [35].
Transforming growth factor (TGF-β)-like class	A vital regulator of embryonic pattern formation. A key regulator of bone induction, maintenance and repair [33].	Marine sponge [36]: *Amphimedon queenslandica* Ctenophore [37]: *Mnemiopsis leidyi* Mollusca [38]: *Planorbarius corneus*,* Viviparus ater*, *Viviparus contectus*, *Lymneae stagnalis*, *Mytilus galloprovincialis*.	TGF-β ligands and TGF signal pathway components (e.g., SMAD) in early development and embryo patterning [39].
Vascular endothelial growth factor (VEGF)	A cell signaling protein that induces vasculogenesis and angiogenesis [39].	Cnidaria: *Podocoryne carnea* [20].	VEGF signal pathway elements involved in morphogenesis of tentacle and gastrovascular canals.
Fibroblast growth factor (FGF)	ECM signaling protein that activates important pathways in skeletal development and regulate chondrogenesis and osteogenesis. A powerful angiogenic factor [40].	Cnidaria: *Nematostella vectensis* [41].	FGF ligands and receptors present where they function in gastrulation and development of chemosensory apical organ of ciliary larvae [42].
Insulin like growth factor (IGF1-IGF2)	Linchpin in stimulating bone formation and remodeling. Regulates chondrocyte growth and metabolism. Stimulates collagen synthesis. Site-directed recruitment of osteoblasts [39].	Nacre seashell: *Haliotis laevigata* [43].	Nacre perlustrin has homology with *N*-terminal domain of mammalian insulin-like growth factor binding proteins (IGFBPs) [43].
Tissue necrosis factor (TNF)	Inflammation cytokine and regulator of immune cells. One role is as a potent actor in bone re-modeling. It carries out this function through the MAPK pathway which, controls differentiation and proliferation through JNK mediator. It is also prominent regulator of osteoclastogenesis [39].	TNF-α present in *Mytilus edulis* [44,45]. Their immunocytes respond to TNF in same manner as human granulocytes.	Cytokine analogues present in *Mytilus edulis* neural tissue [44,45].
Epithelium growth factor (EGF)	A vital regulator of bone cell metabolism in formation and resorption [46].	Marine sponge: *Lubomirskia baicalensis* [47].	Genes coding for EGF-like molecules expressed for patterning silica structural modules
Platelet derived growth factor (PDGF)	A keystone regulator of cell division and growth. It is vital inductive signal in tissue remodeling morphogenesis and cell differentiation. It is a potent mitogen for bone cells. It also functions as a chemotactic factor for MSC and osteoblasts [48].	Mollusca [48] *Planorbarius corneus*, *Viviparus ater*, *Viviparus contectus*, *Lymneae stagnalis*, *Mytilus galloprovincialis*.	Stimulate chemotaxis and phagocytic activity of wound repairing Molluscan immunocytes [48].

**Table 2:** Proteins separated from organic matrices in coral skeletons by molecular weight.

Coral species	MW banding in kDa	Reference	Amino acid compositions	Protein gel chromatography
Synularia polydactyla (spicules)	109, 83, 70, 63, 41, 30, 22	Rehman *et al.* 2005 [49].	Sequencing of 70 kDa and 63 kDa proteins. Enriched acidic amino acids. 70: glutamate; 63: glycine.	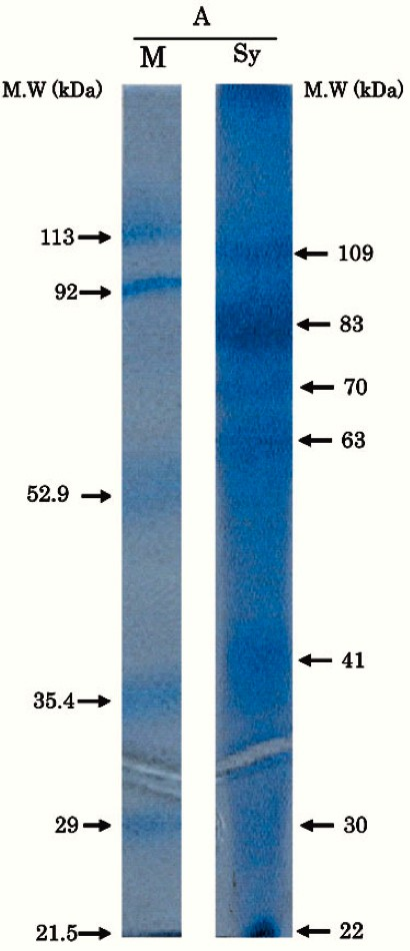 Reproduced with permission from Wiley-VCH Verlag GmbH, 2005 [49].
Galaxea fascicularis	53, 45	Fukuda *et al.* 2003 [50].	Rich in cysteine (dicysteine repeat pattern) Aspartate and asparagine.	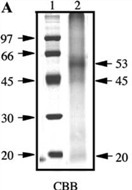 Reproduced with permission from Elsevier Inc., 2003 [50].
Balanophylli a europaea	IOM and SOM: 14.4, 13.9, 66	Goffredo *et al.* 2011 [51].	SOM: high acidic amino acids. IOM: high hydrophobic residues.	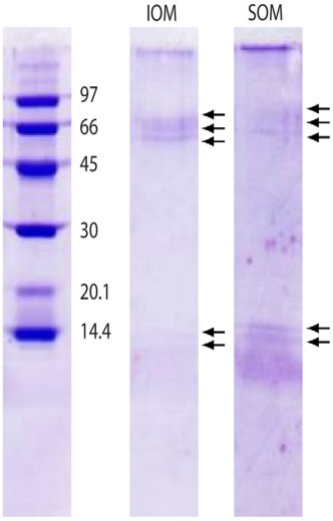 Reproduced with permission from *PLoS One* [51].
Stylophora Pistillata Pavona Cactus	55, 37, 47, 68, 50, 47, 37, 33 (from column 1: silver staining)	Puverel *et al.* 2005 [52].	SOM: Soluble organic matrix.	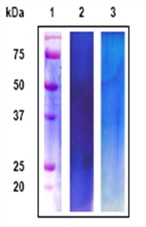 Reproduced with permission from Elsevier Inc., 2005 [52].
C. rubrum	(A): 6 bands at 81, 55, 47, 44, 12 and 10 kDa;	Deubreil *et al.* [53].		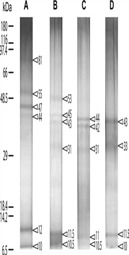 Reproduced with permission from Springer 2011 [53].
C. konojoi	(B): 6 bands at 53, 45, 43, 31, 11.5 and 10.5 kDa;			
C. secundum	(C): 5 bands at 44, 42, 31, 11 and 10.5 kDa;			
C. elatius	(D): 4 bands at 43, 33, 11.5 and 10 kDa.			

**Table 3:** Proteins separated from organic matrices in marine sponges by molecular weight.

Marine sponge species	MW banding in kDa	Reference	Amino acid compositions
Astroscleria willeyana (A coralline demosponge)	33–41	Jackson *et al.* 2006 [54]	Not characterised

We can be increasingly confident that the approach, to harness proteins from marine invertebrate skeletons will be beneficial for strategies in bone regeneration. This is because the best evidence yet, came to light, when nacre organic matrix proteins were shown to instigate human bone formation. 

### Nacre Organic Matrix Proteins Can Promote Bone Formation in Humans

Proteins derived from the skeletal organic matrix of nacre seashell have been shown, repeatedly, to promote the skeletal building activities of human bone cells, both in the culture dish and in a patient*.* The pioneering surgery only happened once. It included nacre particles, which were used to plug and seal a mandibular bone void. The nacre promoted regeneration of new bone contiguous with existing native bone [55]. Extra studies incorporating bone cells with nacre, in the petri-dish, provided evidence that the observed osteoinduction was probably due to the release of a mobile cell signaling peptide, eminating from the skeletal organic matrix [56]. Proteins extracted and then separated from the organic matrix showed similarities with bone growth peptides. To test this notion concentrated extracts of the skeletal organic matrix were infused into media bathing un-specialized cultured cells and resulted in their trans-differentiation into osteoblast like cells. The active soluble matrix fraction was found to consist of low molecular weight molecules. Using size exclusion and anion exchange HPLC, four defined fractions were identified, enriched in alanine and glycine. According to Almeida *et al.* [57] molecules present in one of these fractions are involved in driving the commitment of cells into the osteoblast lineage which is closely related to the TGF-β molecular superfamily of growth factors and BMP, because of the correspondence in biochemical responses of treated bone cells *in vitro* to those same molecules [58,59]. So far, there is no conclusive data to match the amino acid sequences of nacre organic matrix peptides and these prominent osteogenic proteins operating in human bone matrix. Very recently four proteins derived from SPM of nacre, distinct from the previous studies in this matter, were identified as having homologous sequences to the bone re-modeling factors, Wnt inhibitor factor (WIF) and tenascin C [60].

## 6. Use of Proteomic Methods to Retrieve Clinically Relevant Matrix Proteins

Proteomics will be an important tool for the discovery, characterization and analysis of medically useful marine derived proteins. Proteome studies begin by applying rigorous fractionation technologies and steps—designed through trial and error optimization—for each sample type and source [61]. These methods provide the most precise evaluation of protein identities, composition, abundance, cell surface modifications and vital function including protein expression profiling [62,63].

The analysis of protein compositions in the organic matrices of many marine invertebrates has been limited because of the difficulties in separating them by molecular weight and acidic range [64]. In a handful of studies there have been surveys of protein molecular weights, isoelectric points (Table 2, Table 3), molecular bonding and amino acid compositions of organic matrices, both soluble and insoluble-mineral bound [63,64,65]. Classical chromatography is often the first step in deriving purified homogenous native proteins and protein fractions in preparation for proteomic analysis. These provide more manageable levels of fractionated proteins from specific regions of the proteome. It is especially important in studies where one needs to maintain the activity of desired protein such as, in marine products drug discovery research programs. One area of major interest for the bone tissue engineer is discovering a set of proteins that can individually and collectively manipulate stem cell proliferation and differentiation. Then use this control to induce osteomorphogenesis accurately and rapidly.

In assessing the cell acting potential of retrieved proteins it is vital that cell-based assays are carried out, such as with mesenchymal stem cells. These are the prime stem cell type used in experimental tissue engineering of bone. The best sequence of steps to follow is first, identify proteins of interest using classical chromatography to group them according to molecular weight and electrical charge, then use them in assays to determine how the stem cells respond and to precisely identify their amino acid composition using sophisticated analytical machines. Chromatography is designed to separate proteins into clearly resolved isolates both before and after cell-based proliferation and differentiation assays have been carried out. Proteins with defined osteoactive roles on stem cell populations can be retrieved following duplication of separation and cell assays. To ensure maximized accuracy of amino acid sequencing the sample has to be eventually fractionated to homogeneity. The composition of the protein is cross-checked with existing databases of human proteins to discover its analogue. It is also perfectly feasible that proteins with osteoactive potency may not have a human counterpart. Stable isotope labeling by amino acids in cell culture (SILAC) and Isobaric tag for relative and absolute quantitation (iTRAQ) tagging methods, together with RNAseq profiling, to catalogue the entire complement of RNA, can be used to prepare a global portfolio of the stem cell proteome, following their treatment with marine invertebrate proteins. When measured serially over time, it then allows us to chart the protein interaction networks generated, in response to the addition of the marine invertebrate proteins. Such an approach provides us with more accurate and realistic information about the status of the treated cell and the nature of its activity.

The application of proteomic analysis and profiling could increase the efficiency of screening new candidate molecules from a large set of extracted proteins and how they are modified after translation. The utility of proteomic analysis will be dictated by how much the original sample retrieved from the organism has to be fractionated to reduce its complexity and the dynamic range of protein concentration available for analysis. The potential to do this has been demonstrated recently in an experimental study to characterize the biochemical make-up of bone matrix proteins [66]. This study demonstrates how it is possible to characterize an entire set of proteins in a nanoscale sample of source bone material. It also lays out a thorough blueprint for examining the biochemical make-up of organic matrices in mineralized tissues even at low levels of abundance—1%–2% [66]. Use of the proteomic techniques just described, in marine product chemistry, has not been extensive and this is also reflected in the paucity of protein databases that exist for these organisms. So far there have been a handful of notable studies that employed a proteomic approach to analyze protein secretions from the skeletal organic matrix of marine organisms, namely on *Nautilus*, Stony coral and Abalone shell [67,68,69]. Probing and analyzing the proteome can reveal surprises. The principal one has been to uncover many new, unknown proteins. For example, 82 entirely new proteins were retrieved from the matrix of sea urchin tooth; the further surprise was that they had specific roles specified by their anatomical location [70].

## 7. Conclusions

Biologists have known that the basic proteins (and sugars such as, GAGs) involved in building human skeletal tissues are also the same types used in building simple marine invertebrate skeletons. In Table 1, we provided a list of evidence showing that major growth factor proteins—with varying degrees of participation in human skeletal formation—and commonly used in tissue engineering strategies, are also present in marine invertebrates. The most prominent regenerative proteins so far identified are TGF-β and BMP-2/4. These molecules play a comprehensive role in development of tissues. The invertebrate analogues are almost identical to the human protein in that they have similar building block compositions and functions. 

Marine invertebrates express a high degree of chemical and structural diversity. This includes a broad range of protein and sugar (GAGs, carbohydrates and lectins) molecules that participate in development, signaling, metabolism and regeneration. These organisms are more accessible models for a laboratory-based study of molecular processes and events during biomineralization. This compared to larger, more complicated orders of invertebrates and vertebrates. It is also feasible to extract enough protein to be able to analyze and sequence effectively using proteomic techniques including mass spectrophotometry. The combined use of chromatography, cell assays and proteomics could provide an effective means of discovering therapeutically significant human analogues. Therefore, the use of simple marine invertebrates could potentially provide the rapid development of low-cost, ready-made osteoinductive molecules as well as structural matrix materials for musculoskeletal tissue engineering to potentially replace and augment the use of some recombinant human growth factors and human ECM molecules. 

Methods for *in situ* marine aquaculture that are more compatible with their host growth requirements will be pivotal in the future to facilitate sustainable, large-scale production of organic matrix protein production. Traditional methods are still the best, because they are easier to implement and have the lowest costs. Locating ideal areas for growing invertebrates along the coast is uniquely challenging. However, tightly controlled environments can be organized for lab-based cultivation of marine invertebrate candidates. Lessons need to be learnt on what constitutes the best growing conditions for each particular species of marine invertebrate. The aim of all this, is to grow marine invertebrates that secrete a specific protein of therapeutic importance. It is also feasible to grow marine invertebrates where genes for a desired protein, of therapeutic importance, are programmed to over express the protein. For the bone tissue engineer the key molecules sought from the skeletal organic matrices and extracellular matrices are analogues of growth factors, calcifying promoters, regulators and adhesive peptides. There needs to be certainty and confidence that the functions of the substitute invertebrate protein are identical to their human counterpart. Some studies have shown that certain proteins from one organism can function normally in another distantly related organism.

A rational argument against exploiting the protein products from marine invertebrates is that the harvested yields can often be too low in quantity to justify the concerted effort necessary for collection, extraction, purification, concentration and biological testing. Judicious selection is necessary to avoid targeting marine invertebrate candidates, which are ecologically significant keystone species or listed as endangered. The use of laboratory-based marine invertebrate cell and tissue explant culture methods, in lab-based mariculture, will eventually circumvent these serious drawbacks. In controlled laboratory environments there is the potential to rigorously control the environment, to promote and regulate secretions of the desired protein. In the longer term this would be more of an advantage than growing whole organisms. However, it has been difficult to establish long-term cell lines and generate ideal growing media to sustain them.

The combined present day effectiveness of mariculture, to cultivate whole marine organisms, marine invertebrate cell culture to generate soluble proteins directly in the culture dish and proteomic analysis will guarantee the most efficient use and maximized potency for marine invertebrate originated regenerative proteins. Crucially, it will maximize the availability of these proteins without damaging and degrading precious coral reef habitats.
